# Inside stent placement is suitable for preoperative biliary drainage in patients with perihilar cholangiocarcinoma

**DOI:** 10.1186/s12876-024-03266-z

**Published:** 2024-05-20

**Authors:** Reiko Yamada, Naohisa Kuriyama, Takamitsu Tanaka, Kenji Nose, Yoshifumi Nakamura, Tetsuro Miwata, Junya Tsuboi, Shugo Mizuno, Hayato Nakagawa

**Affiliations:** 1 Gastroenterology and Hepatology, Mie University Graduate School of Medicine, 2-174, Edobashi, Tsu, Mie-prefecture 514-8507 Japan; 2 Hepatobiliary Pancreatic and Transplant Surgery, Mie University Graduate School of Medicine, Tsu, Japan

**Keywords:** Endoscopic biliary stenting, Inside stent, Localized perihilar cholangiocarcinoma, Preoperative biliary drainage, Time to recurrent biliary obstruction

## Abstract

**Background/Purpose:**

Endoscopic biliary stenting (EBS) is commonly used for preoperative drainage of localized perihilar cholangiocarcinoma (LPHC). This study retrospectively compared the utility of inside stent (IS) and conventional stent (CS) for preoperative EBS in patients with LPHC.

**Methods:**

EBS was performed in 56 patients with LPHC. EBS involved the placement of a CS (*n* = 32) or IS (*n* = 24). Treatment outcomes were compared between these two groups.

**Results:**

Preoperative recurrent biliary obstruction (RBO) occurred in 23 patients (71.9%) in the CS group and 7 (29.2%) in the IS group, with a significant difference (*p* = 0.002). The time to RBO (TRBO) was significantly longer in IS than in CS (log-rank: *p* < 0.001). The number of stent replacements was significantly lower in IS than CS [0.38 (0–3) vs. 1.88 (0–8), respectively; *p* < 0.001]. Gemcitabine-based neoadjuvant chemotherapy (NAC) was administered to 26 patients (46.4%). Among patients who received NAC, TRBO was longer in IS than in CS group (log-rank: *p* < 0.001). The IS group had a significantly shorter preoperative and postoperative hospital stay than the CS group (20.0 vs. 37.0 days; *p* = 0.024, and 33.5 vs. 41.5 days; *p* = 0.016).　 Both the preoperative and the postoperative costs were significantly lower in the IS group than in the CS group (*p* = 0.049 and *p* = 0.0034, respectively).

**Conclusion:**

Compared with CS, IS for preoperative EBS in LPHC patients resulted in fewer complications and lower re-intervention rates. The fact that the IS group had shorter preoperative and postoperative hospital stays and lower costs both preoperatively and postoperatively compared to the CS group may suggest that the use of IS has the potential to benefit not only the patient but also the healthcare system.

**Supplementary Information:**

The online version contains supplementary material available at 10.1186/s12876-024-03266-z.

## Background

For localized perihilar cholangiocarcinoma (LPHC), surgical resection is the only curative treatment that increases the opportunity for long recurrence-free survival. During the past two decades, advances in diagnostic and surgical techniques have improved surgical outcomes and survival rates [1]. In addition, appropriate preoperative biliary drainage is required for surgical planning because most patients with LPHC have jaundice and liver dysfunction. Thus, preoperative biliary drainage can be essential to reduce postoperative complication rates and mortality in patients requiring extensive hepatectomy [2, 3].

Preoperative endoscopic biliary stenting (EBS) is a useful drainage method in patients with PHC accompanied by obstructive jaundice and/or cholangitis. Methods of biliary drainage include percutaneous transhepatic biliary drainage (PTBD), endoscopic nasobiliary drainage (ENBD), and EBS. PTBD has risks of portal and hepatic artery injury during puncture as well as fistula recurrence [4–6]. Thus, PTBD is generally not the first choice for biliary drainage. ENBD has the advantage of facilitating observation of the amount and properties of the bile and has a low risk of cholangitis development [7]; additionally, preoperative drainage by ENBD is recommended in the Japanese guidelines [2]. However, ENBD has the disadvantages of causing physical and emotional discomfort to the patient (e.g., because of the need for bile replacement); causing discomfort in the nasal passage and pharynx; and the need for hospitalization.

Plastic stents (PSs) are usually used for preoperative EBS in patients with malignant perihilar biliary stricture. PS occlusion occurs as a result of duodenobiliary reflux and bacterial adherence to the inner wall of the stent, leading to formation of sludge [8, 9]. The sphincter of Oddi carries out an important function by blocking reflux of duodenal juice, which contains bacteria. Researchers have investigated the use of an inside stent (IS), which places the PS above the sphincter of Oddi, to retain this bacteriological barrier and prevent reflux cholangitis [10]. Some reports have stated that ISs are useful for preventing reflux cholangitis following liver transplantation [11, 12].

Several recent reports have addressed the utility of IS placement for malignant biliary strictures. Uchida et al. [13] reported that an IS showed a better patency period than a conventional stent (CS) placed across the papilla of Vater in patients with unresectable malignant biliary stricture. Kobayashi et al. [14] reported that an IS was used for preoperative drainage in patients with biliary tract cancer, and the occlusion rate was significantly lower than that of a CS. Nakamura et al. [15] also reported that an IS has a lower re-intervention rate and relatively long-term patency during the preoperative period; they also found that an IS was useful for preoperative biliary drainage in patients with malignant perihilar biliary stricture, especially those requiring a long drainage period before surgery.

This study was performed to retrospectively compare the results of IS and CS placement in patients with LPHC and to clarify the utility of an IS for preoperative drainage.

## Methods

This retrospective study was approved by our institution’s ethics committee (Mie University Hospital, Clinical Research Approval No. H2021-195). In this retrospective study, the Ethics Committee approved that informed consent for each patient was not required and opt outs were made. The study was conducted in accordance with the principles of the Declaration of Helsinki. The patients’ clinical information was extracted from a maintained database at the Department of Hepatobiliary Pancreatic and Transplant Surgery, Mie University Hospital, and verified by reviewing patient medical records. The day of final follow-up was 31 May 2023.

### Patients

**Fig. 1:**
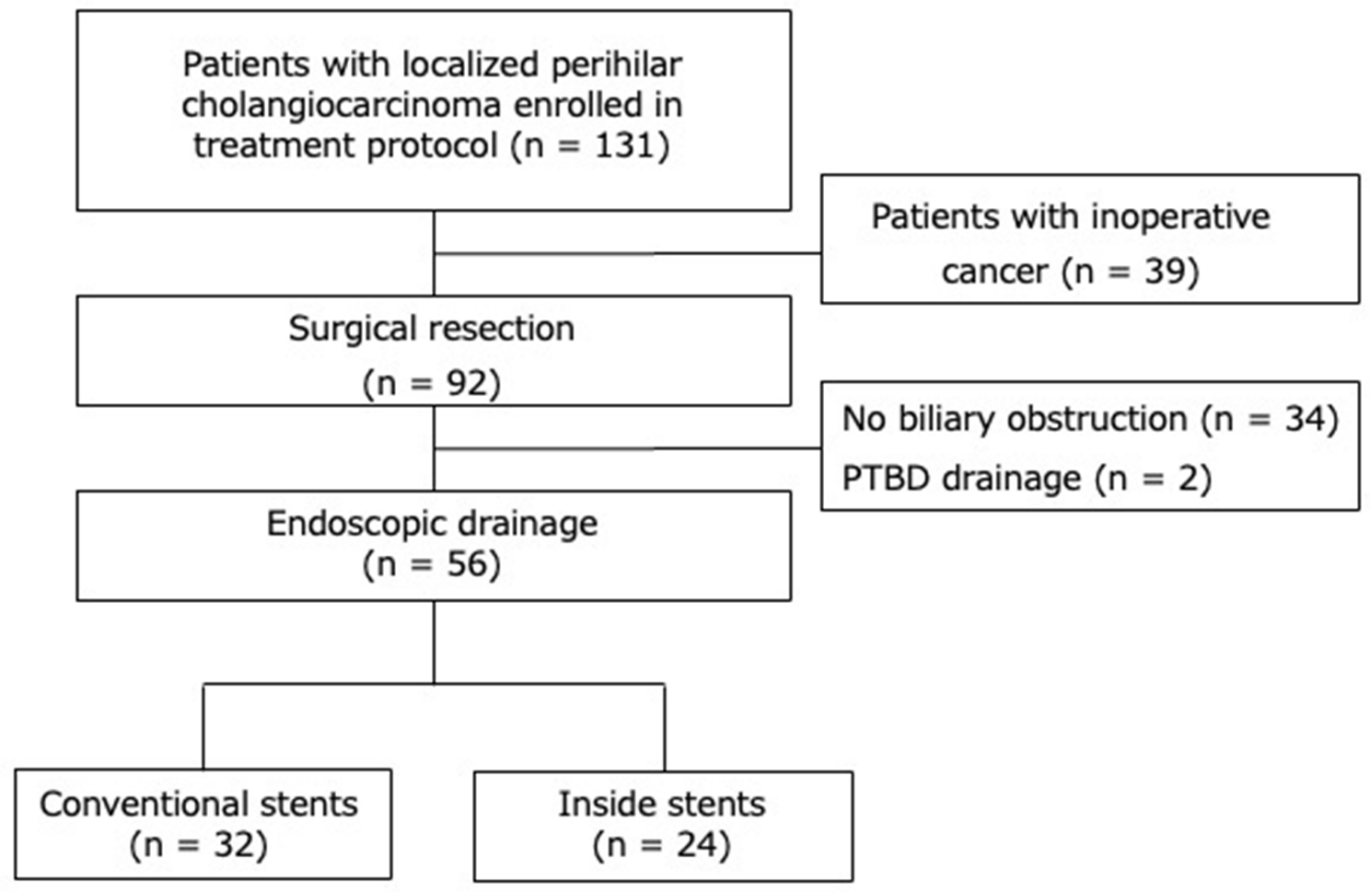
Flow diagram of the study participants. PTBD: percutaneous transhepatic biliary drainage

From January 2011 to December 2022, 131 consecutive patients with LPHC were enrolled in a treatment protocol at Mie University. All patients had undergone computed tomography (CT) examinations and been diagnosed with primary biliary tract carcinoma by expert radiologists. The diagnosis of LPHC was confirmed by cytological analysis of bile juice or histological analysis of biopsy specimens obtained using endoscopic retrograde cholangiopancreatography (ERCP). Patients who showed evidence of distant metastatic lesions at the time of enrollment were excluded from the study. A total of 92 patients underwent surgical resection, and all patients were pathologically diagnosed with primary PHC. According to the resectability classification based on intraoperative biliary and vascular factors established by our team, we added preoperative chemotherapy to the treatment of patients with LPHC. We excluded 34 patients who had not undergone biliary drainage and 2 patients who had undergone PTBD as the initial treatment. Ultimately, 56 patients who underwent EBS were retrospectively enrolled in the present study (Fig. 1).

There are two main strategies used for EBS during the waiting time before definitive surgery. (1) Single EBS was planned before surgery. In this situation, the initial EBS is considered as the last planned EBS. It shall be defined as the “single EBS”. (2) In cases that investigations such as biopsy and cholangioscopy were not performed during the initial procedures, and then performed in a subsequent planned EBS procedures, the subsequent procedure shall be defined as the “subsequent EBS”.

### Preoperative endoscopic treatment (stent placement)

**Fig. 2:**
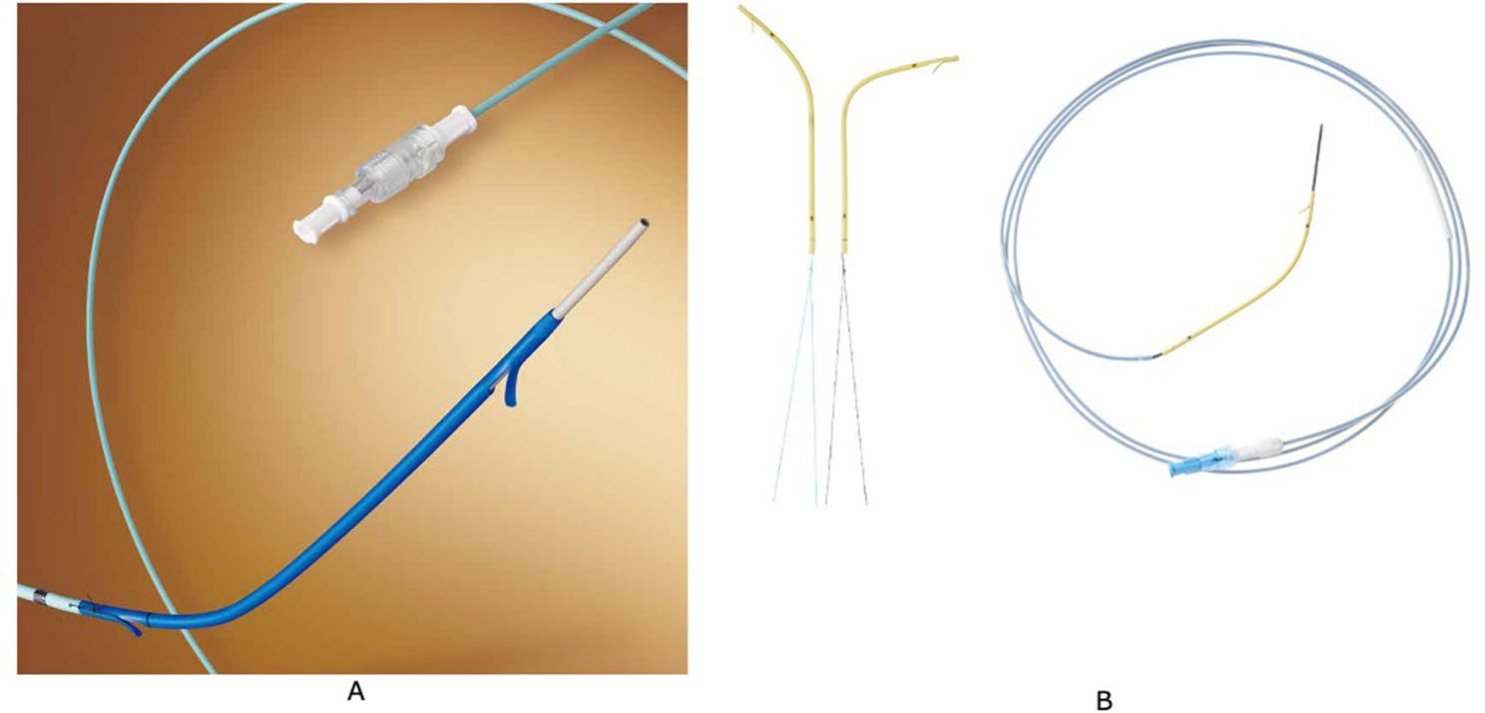
(**A**) The conventional stents used in this study. 7-Fr or 8.5-Fr Amsterdam-type polyethylene stents (Flexima™ Biliary Stent; Boston Scientific). (**B**) The inside stents used in this study. An Amsterdam-type polyethylene stent (Through & Pass™ IS; Gadelius Medical K.K., Tokyo, Japan). There are two types of central bends, light angle (left) and deep angle (right)

The initial resectability classification was evaluated based on dynamic multidetector-row CT (MDCT) findings before biliary drainage. The detailed diagnoses were established by ERCP using a video-duodenoscope (JF260V or TJF260V; Olympus Medical Systems, Tokyo, Japan) and intraductal ultrasonography or a digital single-operator cholangioscope (SpyGlass™ DS, or SpyScope™ DS II; Boston Scientific, Natick, MA, USA). We evaluated the site and type of biliary stricture according to the Bismuth classification [16]; selective cannulation under ERCP was performed for segmental duct evaluation. Endoscopic sphincterotomy (EST) was performed to confirm the diagnosis by histological analysis of biopsy specimens in almost all patients. After diagnostic biopsy, biopsies of the root of the posterior bile duct, root of B4, and bifurcation of B2 and B3 were performed to obtain histological evidence of biliary extension for surgical planning. Finally, endoscopic retrograde biliary drainage tubes were inserted in patients with obstructive jaundice. For drainage, we used a CS (placed across the sphincter of Oddi) or an IS (placed above the sphincter of Oddi). Multiple stents were inserted if clinically indicated. The CSs used in this study were 7-Fr or 8.5-Fr Amsterdam-type polyethylene stents (Flexima™ Biliary Stent; Boston Scientific) (Fig. 2A). The ISs used in this study were an Amsterdam-type polyethylene stent (Through & Pass™ IS; Gadelius Medical K.K., Tokyo, Japan) (Fig. 2B) or the Advanix™ J stent (Boston Scientific). In our hospital, CSs have been used since 2011, the year in which the present evaluation started; ISs have been gradually used since late 2014, and in recent years, ISs have been used more frequently. The stent size used in this study was either 7 or 8.5 Fr, and the length was 9–12 cm. A central bend with either a light or deep angle was selected according to the bile duct configuration. For patients with cholangitis at the time of recurrent biliary obstruction (RBO) during the preoperative period, the stents were replaced. These treatments were repeated until surgical resection.

### Resectability classification of LPHC and preoperative treatment

Among all 56 patients, 26 (46.4%) underwent preoperative chemotherapy. The study protocol of this preoperative treatment was approved by the medical ethics committee of Mie University Hospital (ID2954, UMIN ID: 000030980). The all patients gave their written informed consent for inclusion in the preoperative chemotherapy. Mie University established an anatomical resectability classification for LPHC with three categories: resectable, borderline resectable, and locally advanced according to biliary and vascular factors (Supple Table 1) [17]. Up-front surgery was performed for patients with resectable disease without clinical evidence of lymph node metastasis based on the findings of PET-CT and MDCT. Neoadjuvant chemotherapy (NAC) was performed for patients with resectable cancer with lymph node metastasis, borderline resectable cancer, and locally advanced cancer (Supple Table 1). Until December 2021, the NAC regimen included two cycles of chemotherapy with gemcitabine (800 mg/m^2^ on days 7 and 21) plus S-1 (80 mg/body daily on days 1–21 every 4 weeks) (GS therapy) [18, 19]. In January 2022, the NAC regimen changed to four cycles of gemcitabine plus cisplatin plus S-1 (GCS therapy) [20].

### Evaluation

We evaluated the preoperative status of patients with or without cholangitis and assessed their laboratory data at the time of pre-endoscopic treatment. RBO was defined as a composite of stent occlusion and stent migration according to the TOKYO criteria 2014 [21]. We defined acute cholangitis according to the Tokyo Guidelines 2018 [22]. We evaluated the number of endoscopic treatments until surgical resection. Post-ERCP complications were also evaluated according to the criteria established by Cotton et al. [23]. Additionally, we evaluated the patients’ hospital stays after surgical resection and the occurrence of severe postoperative complications using the Clavien–Dindo grading classification [24].

### Statistical analysis

Continuous and categorical variables were expressed as median (range) and were compared using the Mann–Whitney test and Fisher’s exact test. Countable variables such as number of stents and number of stent replacements were expressed as average (range) and were compared using the Student’s t-test. Adverse events were graded according to the literature [25]. In all patients who returned for reassessment, the date of the initial endoscopic drainage was chosen as the starting point for measurement of the time to RBO (TRBO). The median cumulative TRBO with the 95% confidence interval was calculated using Kaplan–Meier analysis and compared using the log-rank test. The day of final follow-up was 31 May 2023. All statistical analyses were performed using SPSS version 29 (IBM Corp., Armonk, NY, USA). A p value of < 0.05 was considered statistically significant.

### Data availability

The datasets used and/or analysed during the current study available from the corresponding author on reasonable request. The authors will provide an email address for communication once the information sharing is approved. The proposal should include detailed aims, statistical plan, and other information/materials to guarantee the rationality of requirement and the security of the data. The related patient data will be shared after review and approval of the submitted proposal and any related requested materials. Of note, data with patient names and other identifiers cannot be shared.

## Results

### Patients’ characteristics

The patients’ characteristics are summarized in Table 1. The CS group (*n* = 32) and IS group (*n* = 24) showed no significant differences in median age or sex. Fifty-six patients were retrospectively enrolled in the current study. All patients underwent surgical resection and were pathologically diagnosed with LPHC.

**Table 1:** Patients’ characteristics and the clinical outcomes of the whole patients

Patients’ characteristics	CS (*n* = 32)	IS (*n* = 24)	*p* value
Age (years)	72 (51–87)	70 (44–85)	0.446
Sex (male/female)	19/13	17/7	0.274
Bismuth III, IV	22 (68.8%)	22 (91.7%)	0.038
EBS plan (single/subsequent)	22/10	12/12	0.126
Cholangitis before initial drainage	0 (0.0%)	0 (0.0%)	N.S.
Endoscopic sphincterotomy	18 (56.3%)	23 (95.8%)	< 0.001
Number of stents (average)	1.25 (1–2)	1.46 (1–2)	0.115
Initial drainage to surgery, days	91 (13–394)	71 (22–289)	0.354
Drainage area (unilateral/bilateral)	24/8	13/11	0.090
Neoadjuvant therapy	15 (46.9%)	11 (45.8%)	0.577
Portal vein embolization	6 (18.8%)	3 (12.5%)	0.402
Major hepatectomy (resection of two or more segments)	23 (71.9%)	22 (91.7%)	0.063
Clinical outcomes			
Preoperative cholangitis	23 (71.9%)	7 (29.2%)	0.002
Number of stent replacements (average)	1.88 (0–8)	0.38 (0–3)	< 0.001
Poor improvement of jaundice	1 (3.1%)	1 (4.2%)	0.678
Adverse events (others)	7 (18.8%)	1 (4.2%)	0.064
Pancreatitis	6 (18.8%)	1 (4.2%)	0.108
Bleeding	1 (3.1%)	0 (0.0%)	0.678
Postoperative adverse events (Clavien–Dindo grade > IIIa)	18 (56.3%)	17 (70.8%)	0.202

Data are presented as median (range), n, or n (%) unless otherwise indicated

CS: conventional stent, IS: inside stent, EBS: endoscopic biliary stenting, N.S.: not significant

The proportion of patients with Bismuth type III and IV stricture was higher in the IS than CS group [22 (91.7%) vs. 22 (68.8%), respectively; *p* = 0.038]. With regard to the EBS plan, single EBS were performed in 22 cases of CS group and in 12 of IS group. Whereas, subsequent EBS were performed in 10 of CS group and 12 of IS group; there was no significant difference between both groups (*p* = 0.126). No cholangitis before initial drainage was found in either group. EST was performed in more patients in the IS than CS group [23 (95.8%) vs. 18 (56.3%), respectively; *p* < 0.001]. The average number of inserted PSs was not significantly different between the CS and IS groups [1.25 (1–2) vs. 1.46 (1–2), respectively; *p* = 0.115]. In addition, no significant difference was found in the drainage area (CS group: unilateral/bilateral = 24/8, IS group: 13/11; *p* = 0.090). Per-cutaneous transhepatic portal vein embolization (PTPE) was performed in six patients (18.8%) in the CS group and three (12.5%) in the IS group, with no significant difference between the two groups (*p* = 0.402). NAC was administered to 15 patients (46.9%) in the CS group and 11 (45.8%) in the IS group, also with no significant difference between the two groups (*p* = 0.577). There was no significant difference in the median waiting time from initial drainage to surgery between the CS and IS groups [91 (13–394) vs. 71 (22–289) days, respectively; *p* = 0.354]. The surgical procedures included right hepatectomy in 16 patients, left hepatectomy in 21, extended right hepatectomy in 1, extended left hepatectomy in 3, hepatopancreatoduodenectomy in 5, extrahepatic bile duct resection in 3, subsegment resection in 3, central bisectionectomy in 1, and pancreaticoduodenectomy in 3. Major hepatectomy, which involves resection of two or more segments, was performed in 45 patients, accounting for 80.4% of the total sample. The incidence of major hepatectomy was not significantly different between the CS and IS groups [23 of 32 (71.9%) vs. 22 of 24 (91.7%) patients, respectively; *p* = 0.063].

### Complications and re-intervention rate of preoperative biliary drainage

**Fig. 3:**
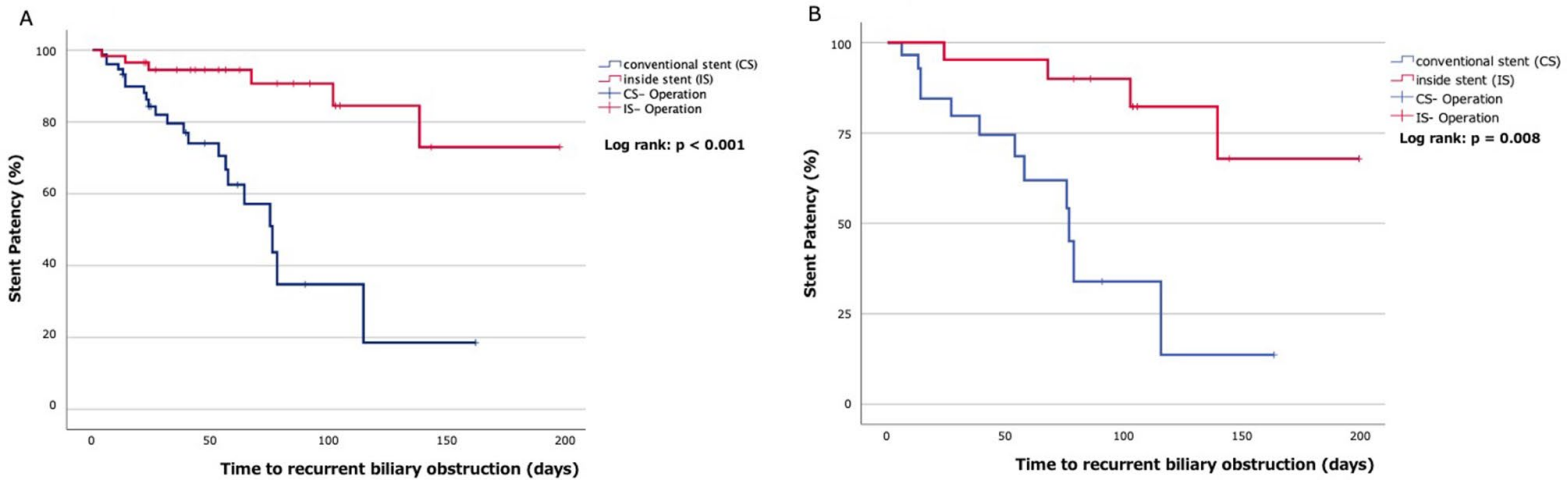
(**A**) Kaplan-Meier curves of recurrent biliary obstruction in the conventional stents and the inside stents of whole patients of the study. (**B**) Kaplan-Meier curves of recurrent biliary obstruction in the conventional stents and the inside stents of patients who was performed neoadjuvant chemotherapy

The incidence of ERCP-related adverse events and the re-intervention rates are shown in Table 1. Preoperative RBO occurred in 23 patients (71.9%) in the CS group and 7 (29.2%) in the IS group, with a statistically significant difference (*p* = 0.002). All patients who developed preoperative RBO underwent bile duct stent replacement. The number of bile duct stent replacements was significantly lower in the IS than CS group [0.38 (0–3) vs. 1.88 (0–8), respectively; *p* < 0.001]. The causes of RBO in the IS group were stent occlusion in six patients and stent migration in one. By contrast, the causes of RBO in the CS group were stent occlusion in 17 patients and stent migration in 6. The TRBO was significantly longer in the IS than CS group (log-rank: *p* < 0.001) (Fig. 3A). In the CS group, 18 patients (56.3%) experienced postoperative adverse events classified above Clavien–Dindo grade IIIa, whereas in the IS group, 17 patients (70.8%) experienced such events. However, no significant difference was observed between the two groups (*p* = 0.202). No deaths occurred during hospitalization in either group.

### Characteristics of patients undergoing NAC and complications of preoperative biliary drainage and re-intervention rates

Gemcitabine-based NAC was administered to 26 patients (46.4%) in this study. In the CS group, 15 patients received GS therapy; in the IS group, 10 patients received GS therapy and 1 received GCS therapy. The characteristics and clinical outcomes were examined in patients who underwent NAC (Table 2). Preoperative cholangitis was observed in 13 (86.7%) patients in the CS group and 5 (45.5%) in the IS group, with a statistically significant difference (*p* = 0.034). The average number of stent replacements was significantly higher in the CS than the IS group [2.73 (range, 0–8) vs. 0.64 (range, 0–3) replacements, respectively; *p* = 0.010]. The TRBO was significantly longer in the IS than the CS group (log-rank: *p* < 0.001) (Fig. 3B). Postoperative complication classified as Clavien–Dindo grade > IIIa occurred in 11 (73.3%) patients in the CS group and 9 (81.8%) in the IS group, with no significant difference between the groups (*p* = 0.491). Among patients who received NAC, the IS group tended to have a longer TRBO and fewer events; the same result was obtained when considering the entire patient cohort.

**Table 2:** Patients’ characteristics and the clinical outcomes of the patients undergoing NAC

Patients’ characteristics	CS (*n* = 15)	IS (*n* = 11)	*p* value
Age (years)	70 (51–87)	71 (62–84)	0.462
Sex (male/female)	10/5	9/2	0.345
Bismuth III, IV	12 (80.0%)	10 (90.9%)	0.426
EBS plan (single/subsequent)	9/6	5/6	0.368
Cholangitis before initial drainage	0 (0.0%)	0 (0.0%)	N.S.
Endoscopic sphincterotomy	7 (46.7%)	11 (100%)	0.004
Number of stents (average)	1.40 (1–2)	1.64 (1–2)	0.252
Initial drainage to surgery (days)	145 (76–394)	145 (79–289)	0.951
Drainage area (unilateral/bilateral)	9/6	4/7	0.214
Portal vein embolization	3 (20.0%)	1 (9.1%)	0.426
Major hepatectomy (resection of two or more segments)	12 (80.0%)	11 (100%)	0.175
Clinical outcomes			
Preoperative cholangitis	13 (86.7%)	5 (45.5%)	0.034
Number of stent replacements	2.73 (0–8)	0.64 (0–3)	0.010
Poor improvement of jaundice	0 (0.0%)	0 (0.0%)	N.S.
Adverse events (others)	4 (26.7%)	0 (0.0%)	0.091
Pancreatitis	4 (26.7%)	0 (0.0%)	0.091
Bleeding	0 (0.0%)	0 (0.0%)	N.S.
Postoperative adverse events (Clavien–Dindo grade > IIIa)	11 (73.3%)	9 (81.8%)	0.491

Data are presented as median (range), n, or n (%) unless otherwise indicated

### Comparison of hospitalization and survival rate

The preoperative and postoperative hospitalization costs and length of hospitalization are summarized in Table 3. The cost of chemotherapy is excluded. The median total duration of hospitalization prior to surgery was 37.0 (range, 1–291) days in the CS group and 20 (range, 1–67) days in the IS group, with a statistically significant difference (*p* = 0.024). In terms of preoperative costs, the CS group had an average total expense of 2,971,491.25 Japanese yen, whereas the IS group had an expense of 1,733,735.42 yen. The CS group incurred significantly higher preoperative costs (*p* = 0.049).

**Table 3:** The preoperative and postoperative hospitalization costs and length of hospitalization

	CS (*n* = 32)	IS (*n* = 24)	*p* value
pre-operative period			
Total hospitalization, days (median, range)	37.0 (1-291)	20.0 (1–67)	0.024
Total medical cost, JPY (average)	2,971,491.25	1,733,735.42	0.049
post-operative period			
Total hospitalization, days (median, range)	41.5 (20–161)	33.5 (16–61)	0.016
Total medical cost, JPY (average)	7,826,488.75	5,696,806.63	0.034

**Table 4:** Comparative studies for preoperative endoscopic transpapillary biliary drainage stenting for malignant hilar biliary obstruction

Year	Autor	Study design	Stent type	number of the patients			pre-operative period (days)			pre-operative period, *P*-value	rate of cholangitis (%)			rate of cholangitis, *P*-value	rate of post proceidure pancreatitis			rate of post proceidure pancreatitis, *P*-value
				CS	IS	ENBD	CS	IS	ENBD		CS	IS	ENBD		CS	IS	ENBD	
2015	Kobayashi, et al. [14]	Retro, single	PS	32	25		96.3	96.8		0.979	46.9%	28.0%		0.15	12.5%	4.0%		0.372
2021	Nakamura, et al. [15]	Retro, single	PS & ENBD	20	41	20	34	33	25	0.89 (IS vs. CS), 0.064 (IS vs. ENBD)	40.0%	9.8%	35.0%	0.013 (IS vs. CS), 0.030 (IS vs. ENBD)	10.0%	4.9%	10.0%	0.592 (IS vs. CS), 0.592 (IS vs. ENBD)
2021	Takahashi, et al. [26]	Retro, single	PS & ENBD		29	49		29	26	0.074		20.6%	40.8%	0.068		6.9%	10.2%	0.621
2023	Ishiwatari, et al. [27]	Retro, multi	PS	56	73		28.5	30		0.41	26.0%	21.4%		0.68	30.1%	25.0%		0.56
2024	Yamada, et al.	Retro, single	PS	32	24		91	71		0.354	71.9%	29.2%		0.002	18.8%	4.2%		0.108

The duration of postoperative hospitalization was 41.5 (range, 20–161) days in the CS group and 33.5 (range, 16–61) days in the IS group. The IS group had a significantly shorter postoperative hospital stay (*p* = 0.016). The postoperative costs were 7,826,488.75 yen in the CS group and 5,696,806.63 yen in the IS group. The IS group had significantly lower postoperative costs (*p* = 0.0034).

**Fig. 4:**
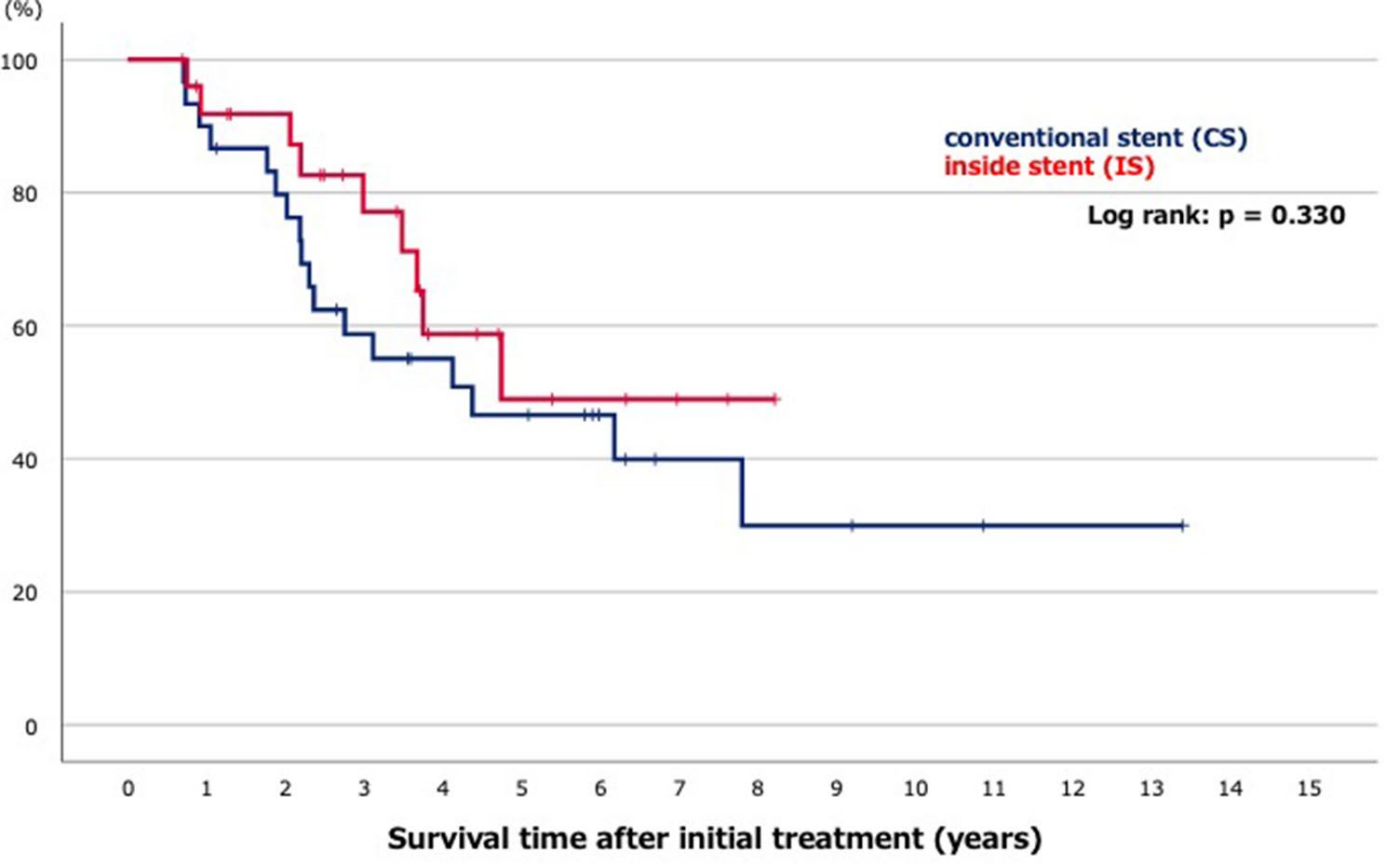
A Kaplan–Meier curve of cumulative patient survival rates between conventional stents and inside stents groups

A Kaplan–Meier curve showed cumulative patient survival rates between CS and IS groups. There was no significant difference in patient survival between CS and IS groups (52.4 vs. 56.8 months, log-rank: *p* = 0.330) (Fig. 4).

## Discussion

In this study, we retrospectively evaluated the outcomes of preoperative EBS in patients with LPHC, specifically the complications and re-intervention rates associated with preoperative biliary drainage. RBO occurred less frequently in the IS than the CS group. The IS group also had a significantly lower number of PS replacements and a longer TRBO. ENBD has been recommended for preoperative biliary drainage in patients with PHC because its risk of cholangitis is lower than in PS placement and there is less dissemination than with PTBD [7]. However, PSs are often used in routine clinical practice because prolonged placement of ENBD tubes impair patients’ quality of life. Recent retrospective studies have suggested that an IS could be an alternative to ENBD in such situations [14, 15, 26]. In addition to the potentially longer TRBO in patients who undergo IS placement, reduced bacterial contamination might lead to a decrease in postoperative infection when an IS is used as preoperative biliary drainage.

Regarding EBS for preoperative PHC with PSs, there have been four retrospective studies (Table 4) [14, 15, 26, 27]. The three studies [14, 15, 26] showed the usefulness of IS as the preoperative EBS. In the study by Kobayashi et al., the median preoperative period was around 3 months, because 47% of patients with malignant PHC received NAC/neoadjuvant chemoradiotherapy [14]. This study showed the significantly lower risk of re-intervention of IS group than CS group. Whereas, Ishiwatari et al. showed that the outcomes of IS and CS for preoperative EBS were comparable for patients with PHC. The preoperative period of their study was around one months. Thus, they considered that the IS might be more favorable for patients with longer waiting time before surgery [27]. Nakamura et al. compared the outcomes of preoperative CS, IS, and ENBD. The re-intervention rate was significantly lower in the IS group than in the CS and ENBD group (9.8% vs. 40% and 35%, *P* = 0.013 and 0.030, respectively) [15]. Takahashi et al. compared the treatment outcome between IS and ENBD [26]. They concluded that IS was a possible alternative to ENBD as a bridge to operation for patients with PHC. In addition, IS insertion was also less likely to cause post-ERCP pancreatitis in all studies. Thus, EBS above the sphincter of Oddi (IS placement) should be selected to avoid pancreatitis in the management of PHC. More RCTs and technical evolutions are expected to result in large changes in the EBS procedure in the future.

Our findings indicate that ISs may offer certain advantages over CSs in patients with PHC, especially those requiring a long drainage period such as NAC and PTPE. IS placement appears to be associated with a lower incidence of preoperative RBO, improved stent patency, and a longer TRBO. These results suggest that IS placement may be a preferable drainage method in the context of NAC. Recent reports have addressed the utility of GS therapy as NAC in patients with pancreatic cancer [28]. In the field of biliary tract cancer, clinical trials are ongoing and NAC for biliary tract cancer will be established in the future [29, 30]. An IS may be suitable as an EBS method for NAC not only because it has a low re-intervention rate but also because it results in a long time to event occurrence.

The IS group had a significantly shorter preoperative and postoperative hospital stay than the CS group (20.0 vs. 37.0 days, respectively; *p* = 0.024 and 33.5 vs. 41.5 days, respectively; *p* = 0.016). The shorter preoperative hospitalization period in the IS group suggests that IS placement might contribute to a more efficient preoperative management process. It is also important to note that there was a significant difference in postoperative costs between the CS and IS groups (*p* = 0.034). Cost reduction can have substantial implications for patients, healthcare providers, and healthcare systems as a whole. Overall, these findings suggest that IS placement in the management of PHC has the potential to optimize resource utilization and decrease healthcare expenditures. Further research is warranted to validate these results and evaluate long-term outcomes, including survival rates and quality-of-life measures, to allow for comprehensive assessment of the clinical and economic benefits associated with IS placement in this patient population.

This study has several limitations, including its retrospective design, small sample size, and use of data from a single institution. These factors may limit the generalizability of the results. Further studies with larger sample sizes and prospective designs are needed to confirm these findings and evaluate long-term outcomes. Nonetheless, the results suggest that preoperative endoscopic treatment with an IS may be a valuable approach in the management of LPHC, potentially improving patient outcomes and optimizing healthcare resource utilization.

In conclusion, use of an IS for preoperative drainage in patients with LPHC resulted in fewer complications and lower re-intervention rates compared with use of an CS; therefore, it may be useful for patients with malignant perihilar biliary stricture, especially those requiring a long drainage period before surgery. Further studies are warranted to validate these findings and explore potential mechanisms underlying the observed benefits.

### Electronic supplementary material

Below is the link to the electronic supplementary material.

Supplementary Material 1

## Data Availability

The datasets used and/or analysed during the current study available from the corresponding author on reasonable request. The authors will provide an email address for communication once the information sharing is approved. The proposal should include detailed aims, statistical plan, and other information/materials to guarantee the rationality of requirement and the security of the data. The related patient data will be shared after review and approval of the submitted proposal and any related requested materials. Of note, data with patient names and other identifiers cannot be shared.

## Abbreviations

EBS: Endoscopic biliary stenting
LPHC: localized perihilar cholangiocarcinoma
IS: inside stent
CS: conventional stent
RBO: recurrent biliary obstruction
TRBO: time to recurrent biliary obstruction
NAC: neoadjuvant chemotherapy
PTBD: percutaneous transhepatic biliary drainage
ENBD: endoscopic nasobiliary drainage
PSs: Plastic stents
CT: computed tomography
ERCP: endoscopic retrograde cholangiopancreatography
EST: Endoscopic sphincterotomy
PTPE: Per-cutaneous transhepatic portal vein embolization
GS: gemcitabine plus S-1
GCS: gemcitabine plus cisplatin plus S-1
