# Supplementary figures and images for: Inside stent placement is suitable for preoperative biliary drainage in patients with perihilar cholangiocarcinoma
Source: BMC Gastroenterol. 2024 May 20;24:174. doi: 10.1186/s12876-024-03266-z (PMC11106890; doi:10.1186/s12876-024-03266-z)

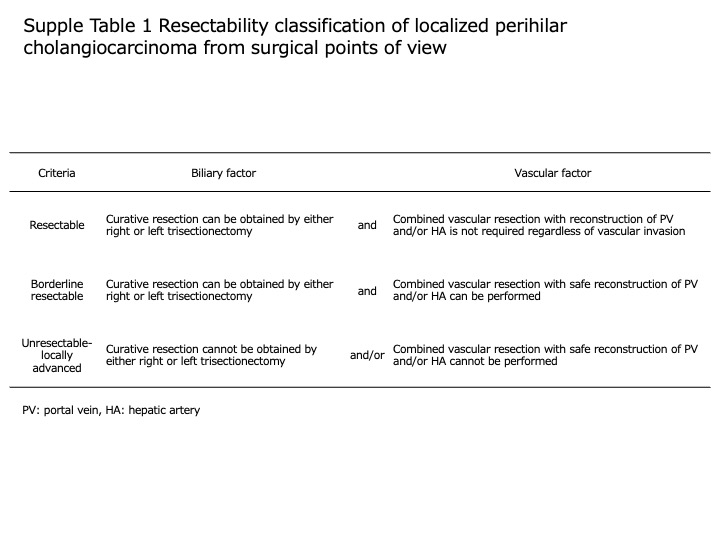

Supplement: Supplementary file 1 — Supplementary Material 1 [file 12876_2024_3266_MOESM1_ESM.jpeg]
